# Swiftly identifying strongly unique *k*-mers

**DOI:** 10.1186/s13015-025-00286-6

**Published:** 2025-07-13

**Authors:** Jens Zentgraf, Sven Rahmann

**Affiliations:** 1 Algorithmic Bioinformatics, Department of Computer Science, Saarland University, Campus E2.1, Saarbrücken, 66123 Saarland Germany; 2 Center for Bioinformatics Saar, Saarland Informatics Campus, Campus E2.1, Saarbrücken, 66123 Saarland Germany; 3 Saarbrücken Graduate School of Computer Science, Saarland Informatics Campus, Campus E2.1, Saarbrücken, 66123 Saarland Germany

**Keywords:** *k*-mer, Hamming distance, Strong uniqueness, Parallelization, Algorithm engineering

## Abstract

**Motivation:**

Short DNA sequences of length *k* that appear in a single location (e.g., at a single genomic position, in a single species from a larger set of species, etc.) are called *unique k-mers*. They are useful for placing sequenced DNA fragments at the correct location without computing alignments and without ambiguity. However, they are not necessarily robust: A single basepair change may turn a unique *k*-mer into a different one that may in fact be present at one or more different locations, which may give confusing or contradictory information when attempting to place a read by its *k*-mer content. A more robust concept are *strongly unique* *k*-mers, i.e., unique *k*-mers for which no Hamming-distance-1 neighbor with conflicting information exists in all of the considered sequences. Given a set of *k*-mers, it is therefore of interest to have an efficient method that can distinguish *k*-mers with a Hamming-distance-1 neighbor in the collection from those that do not.

**Results:**

We present engineered algorithms to identify and mark within a set *K* of (canonical) *k*-mers all elements that have a Hamming-distance-1 neighbor in the same set. One algorithm is based on recursively running a 4-way comparison on sub-intervals of the sorted set. The other algorithm is based on bucketing and running a pairwise bit-parallel Hamming distance test on small buckets of the sorted set. Both methods consider canonical *k*-mers (i.e., taking reverse complements into account) and allow for efficient parallelization. The methods have been implemented and applied in practice to sets consisting of several billions of *k*-mers. An optimized combined approach running with 16 threads on a 16-core workstation yields wall times below 20 seconds on the 2.5 billion distinct 31-mers of the human telomere-to-telomere reference genome.

**Availability:**

An implementation can be found at https://gitlab.com/rahmannlab/strong-k-mers.

## Introduction

Alignment-based biological sequence analysis methods are increasingly being replaced by alignment-free, or at least partially alignment-free, methods. One reason behind this development is the relatively high computational cost for sequence alignments. An early example of this development was the replacement of overlap-consensus based genome assembly by DeBruijn graph based assembly methods, subdividing the sequenced DNA fragments further into overlapping pieces of length *k*, so-called *k*-mers [1]. Another example is pseudo-alignment based transcript quantification from RNA-seq data, pioneered by kallisto [2], which assigns a transcript to each read not by computing alignments, but directly from the *k*-mer content of the reads.

More recently, an alignment-free solution was published for xenograft sorting [3], where one wants to separate reads in a mixed sample from two species; a typical application is to separate human tumor DNA reads from mouse tissue DNA reads in patient-derived xenograft experiments. This had previously been done by aligning all reads to both the human genome and the mouse genome and picking the better alignment for each read to assign the species of origin. However, it is computationally much more efficient to build a *k*-mer index (with $k\ge 25$) with associated species information and to classify reads according to their *k*-mer content. This approach can save up to 80% of the CPU work [3]. Similar ideas generalize to metagenomic profiling, where one seeks to quantify the amount of different species in a metagenomic sample, again not based on alignments, but based on *k*-mer content [4–6].

More examples could be mentioned, but here we want to focus on an important but so far underappreciated aspect of alignment-free methods: the concept of *strongly unique* *k*-mers, as opposed to (weakly) unique *k*-mers and non-unique *k*-mers.

Given a collection of sequences, a *k*-mer is called *unique* if it occurs only once as a length-*k* substring in a single sequence within the collection. Unique *k*-mers are both more useful and technically easier to handle than non-unique *k*-mers: They unambiguously identify a single sequence and a single position within the entire collection. They also allow us to store some information (a “value”) associated with the *k*-mer (the “key”) in a simple way, because there is only a single value for a unique *k*-mer, whereas for non-unique *k*-mers, we would need to handle a variable-length list of values.

Uniqueness, as useful as it is, is not a robust property: A single nucleotide change, such as a sequencing error or an individual single-nucleotide variant, may turn a unique *k*-mer *x* into a different *k*-mer $x'$ that may either not exist at all in the indexed collection, or exist as a unique *k*-mer somewhere else, or as a non-unique *k*-mer in several other locations. The first case (non-existence of $x'$) is usually not so critical; in any case, one knows that there has been a modification. The other cases are more problematic, because an existing *k*-mer was transformed into an equally existing *k*-mer $x'$ that however is (in terms of its location in the collection) not related to $x'$ and could be anywhere or in multiple places.

Therefore, a stronger concept than just uniqueness is helpful. Consider a *k*-mer *x* and its Hamming-distance-1 neighborhood *N*(*x*) containing exactly the 3*k* *k*-mers that differ from *x* at exactly one position. If none of the $x'\in N(x)$ exists in the indexed collection, then *x* is unique with a “safety margin” around it; no single change can turn *x* into an $x'$ that might confuse us with wrong information. We call such *k*-mers *strongly unique*. Strong uniqueness is a useful concept for alignment-free sequence analysis for the reasons stated above: If a strongly unique *k*-mer is seen in a sequenced DNA fragment, that fragment can be unambiguously and robustly located within the indexed collection; a single substitution cannot give wrong information.

A technical complication arises from the double-strandedness of DNA and from the equivalence of a sequence *s* and its reverse complement. Formal details and definitions are therefore presented in Section Preliminaries.

The above explanation should sufficiently motivate us to consider the *weak k-mer identification problem*, formally stated as Problem 1 in Section Preliminaries: Given a *k*-mer set *K*, identify those elements of *K* (called weak *k*-mers) that have a Hamming-distance-1 neighbor in *K*.

After the formal preliminaries, we present two different efficient algorithms for the weak *k*-mer identification problem and an engineered hybrid form (Section Methods). Benchmarks follow in Section Benchmarking Results, and some results on strongly and weakly unique *k*-mers in the human genome in Section Uniqueness within the Human Genome. We discuss two applications that may benefit from our new algorithms (sequencing error correction and xenograft sorting) in Section Application Perspectives, and end with a concluding discussion (Section Discussion and Conclusion).

This article is an extended version of preliminary work published at Wonderful Algorithms in Bioinformatics 2024 [7]. We here additionally evaluate artificial extremal datasets that only contain weak or strong *k*-mers. Our benchmarking results are further enhanced by a more detailed consideration of the interval length threshold at which we switch to pairwise comparisons when running the FourWay algorithm, and we explore refined parallelization by distributing the overall task into many small chunks (Section Benchmarking Results). Beyond benchmarks, we have added detailed insights into the distribution of strongly and weakly unique *k*-mers across the human genome (new Section Uniqueness within the Human Genome). In addition, we discuss potential applications of our algorithms in other alignment-free methods, such as DNA sequencing error correction or xenograft sorting (new Section Application Perspectives).

## Preliminaries

We introduce several basic definitions: *k*-mers, their (canonical) integer encoding, bit-parallel computation of the Hamming distance between two *k*-mers, canonical Hamming distance, and strong and weak *k*-mers in a set.

We only consider DNA sequences over the alphabet $\Sigma =\{\texttt {A}, \texttt {C}, \texttt {G}, \texttt {T}\}$ here (and in our current implementation), but the ideas generalize to other alphabets. However, we take the double-stranded nature of DNA molecules into account when considering Hamming distance between DNA sequences; this would and should not be done with different alphabets.

### Definition 1

*(k-mer)* Given an alphabet $\Sigma$, a *k-mer* is a sequence of length *k* over $\Sigma$. Given a (long) string *s* over $\Sigma$. A *k-mer of s* is any substring of length *k* of *s*.

### Definition 2

*(reverse complement)* The *reverse complement* *rc*(*s*) of a DNA sequence *s* is obtained by reversing the sequence and substituting $\texttt {A} \leftrightarrow \texttt {T}$ and $\texttt {C} \leftrightarrow \texttt {G}$.

### Canonical integer encoding of *k*-mers

To represent and store a DNA *k*-mer *x* efficiently, it can be bijectively encoded as an integer $0\le enc(x) < 4^k$ for fixed *k*: Each base is encoded as a number in $\{0,1,2,3\}$ (e.g. lexicographically), and the resulting sequence of *k* numbers is interpreted as a base-4 integer with *k* “digits”. Equivalently, and relevant for the bit-parallel Hamming distance test described below, we may write the same integer in its 2*k*-bit representation. For example, $enc(\texttt {TACG}) = (3012)_4 = (11|00|01|10)_2 = 198$.

To ensure that a *k*-mer *x* and its reverse complement *rc*(*x*), which both represent the same DNA molecule, are encoded by the same integer value, we define the *canonical integer encoding* or *canonical code* of *x* as $cc(x):= \max \{enc(x), enc(rc(x))\}$. (In most of the literature, the definition is given with the minimum instead of the maximum; this does not matter. Both are equivalently valid ways to assign the same integer to both *k*-mers x and *rc*(*x*).) For example, $cc(\texttt {TACG}) = cc(\texttt {CGTA}) = \max \{enc(\texttt {TACG}), enc( \texttt {CGTA})\} = \max \{198, 108\} = 198$.

### Bit-parallel Hamming distance computation between two *k*-mers

The Hamming distance *d*(*x*, *y*) between two *k*-mers *x*, *y* is the number of positions in which *x* and *y* differ. For 2-bit encoded DNA sequences, there is a fast bit-parallel way to compute the Hamming distance and to test whether $d(x,y) \le 1$.

Let $p = (p_{2k-1},\dots ,p_{0})$ and $q = (q_{2k-1},\dots ,q_0)$ be the 2*k*-bit patterns of *x* and *y*, respectively. From *p* and *q*, we compute a bit pattern $h = (h_{2k-1},\dots ,h_0)$ that has $h_i = 0$ for all odd *i*, and $h_i = 1$ for even *i* if and only if the nucleotides encoded by $(p_{i+1}, p_i)$ and $(q_{i+1}, q_i)$ differ.

To achieve this, we first compute $u:= p \oplus q$, where $\oplus$ is the bitwise exclusive-or (XOR) operation, setting those bits $u_i=1$ where $p_i \ne q_i$. We then combine the two bits of each nucleotide into the even bits of *u* and clear the odd bits to indicate which nucleotides differ between *x* and *y* by setting $h:= (u \,|\, (u \gg 1)) \, \& \, (0101\dots 01)_2$. Here, the operators |, & and $\gg$ represent bitwise or, bitwise and, and bit shift right, respectively. The obtained *h* has the desired properties. The population count (number of 1-bits) of *h* then equals the Hamming distance between the *k*-mers *x* and *y*.

Testing whether the Hamming distance is at most 1 (i.e., whether *h* is zero or a power of 2) can be further simplified by computing $w:= h \, \& \, (h-1)$. We have $w=0$ if and only if *h* has at most a single 1-bit, and $w\ne 0$ if and only if the Hamming distance between the *k*-mers represented by *x* and *y* is at least 2.

### Canonical Hamming distance

In the following, we interpret DNA *k*-mers as double-stranded molecules, i.e., both *x* and *rc*(*x*) are represented by either of them. This has to be considered for Hamming distance computations.

For example, take $x=\texttt {AAAA}$ and $y=\texttt {ATTT}$. Seen as single-stranded *k*-mers, we have $d(x,y)=3$. However, seen as double-stranded DNA molecules, $\texttt {ATTT}$ is equivalent to its reverse complement $\texttt {AAAT}$, and $d(x, rc(y))=1$. Therefore, we make the following definition.

#### Definition 3

*(Canonical Hamming distance)* Given DNA *k*-mers *x*, *y*, their *canonical Hamming distance* is

1 $$\begin{aligned} H(x,y) := \min \{d(x,y), d(x,rc(y))\} \,. \end{aligned}$$

### Problem statement

As motivated in the introduction, our goal is to identify the strongly unique canonical *k*-mers in a collection of sequences. The uniqueness property is identified by *k*-mer counting, for which there exist many efficient methods and tools with different strength and weaknesses, such as KMC2 [8], KMC3 [9], Gerbil [10], Jellyfish [11], hackgap [12], or Kaarme[13], among others. In fact, being able to count up to 2 is sufficient for the present purpose. The output of a *k*-mer counter is a set *K* of distinct canonical *k*-mers, and a count value for each $x\in K$. The missing piece is to identify the weak canonical *k*-mers in *K* in the following sense.

#### Definition 4

*(Weak k-mer, strong k-mer)* Given a set *K* of distinct canonical DNA *k*-mers, a canonical *k*-mer $x\in K$ is called *weak* within *K* if there exists another $y\in K$ with $H(x,y)=1$. The other canonical *k*-mers are called *strong* within *K*.

When we mark weak canonical *k*-mers in *K* and additionally have their counts, we have identified the strongly unique canonical *k*-mers as those with a count of 1 that are not weak in *K*. Therefore, we here focus on the following problem.

#### Problem 1

(Weak canonical *k*-mer identification) Given a set *K* of canonical DNA *k*-mers, identify the subset $W\subseteq K$ of the weak canonical *k*-mers.

How a solution to this problem is implemented in a concrete setting may vary with the representation of *K*. In practice, the elements of *K* will typically be canonical integer codes of *k*-mers, and we may use one more bit to indicate the weak ones.

We point out two obvious algorithms (that are too inefficient in practice) to solve the problem. Let *K* contain *n* distinct *k*-mers.

1. Full pairwise comparison: Use the bit-parallel method from above to test all $$\left( {\begin{array}{c}n\\ 2\end{array}}\right) = n(n-1)/2$$ pairs $x\ne y$ whether they satisfy $H(x,y) \le 1$, and if yes, mark both *x* and *y* as weak.
2. Neighborhood generation: If *K* is given as a hash table allowing efficient look-up, for each $x\in K$, generate the canonical integer codes of the 3*k* Hamming-distance-1 neighbors, and look them up in *K*. For each neighbor found in *K*, mark both *x* and the discovered neighbor as weak.

The first method is impractical because of its quadratic running time. The second method has a running time linear in *n*, but with a factor of 3*k*, and every memory lookup is likely a cache miss. It is thus slow in practice, but can still be useful as a baseline for comparison. In the following section, we present more efficient practical methods for identifying weak canonical *k*-mers.

## Methods

We present two algorithms to identify weak *k*-mers within a set *K*. Independently of the given form of input (hash table based key-value store, or sorted or unsorted list of distinct *k*-mers) and independently of the used algorithm, we first create a *lexicographically sorted* list of the *k*-mers *and* their reverse complements. If the original input consists of canonical *k*-mers, or if the reverse complements are not included, this step may double the input size when we add the reverse complements explicitly. This step is necessary for both algorithms to ensure that we consider the canonical Hamming distance instead of the (standard) Hamming distance for the original input. Details concerning this pre-processing step are given in Section Pre- and Post-Processing after a detailed description of the algorithms.

The first algorithm is based on 4-way comparisons, similar to a multi-way mergesort. In the first round, the sorted *k*-mer list is divided into 4 buckets based on the *k*-mers’ first nucleotide (A, C, G, T). For each nucleotide *c*, a pointer $p_c$ initially points to the start of the *c*-bucket. In each step, we compare the four (or possibly less, once entire buckets have been processed) elements pointed to by the $p_c$, $c\in \Sigma$, ignoring the bucket prefixes. (In the first round, the bucket prefixes are single nucleotides, so we compare the remaining $(k-1)$-mers.) We identify the set $C^*\subseteq \Sigma$ such that the $p_c$, $c\in C^*$, point to the minimal element(s) of the examined $(k-1)$-mers. If $|C^*|=1$, there is a unique minimal $(k-1)$-mer, but if $|C^*|\ge 2$, we have found equal minimal $(k-1)$-mers, i.e. *k*-mers that differ only in their first nucleotide, and therefore a group of weak *k*-mers. The $p_c$, $c\in C^*$, are incremented, and the process is repeated until all buckets have been fully processed. In the next round, each of the buckets is subdivided into 4 sub-buckets, and the 4-way comparison is repeated recursively for each sub-bucket (with *k* decreased by 1). Details are given in Section Recursive 4-way comparisons (FourWay).

The second algorithm does pairwise Hamming distance tests, but on small buckets. If $H(x,y)=1$, then the one difference must be either in the first, second, third, or fourth quarter of the sequence (rounding the boundaries arbitrarily for now). Because the input contains all reverse complements, it is sufficient to consider the third and the fourth quarter: If *x*, *y* differ in the first (second) quarter, then *rc*(*x*), *rc*(*y*) differ in the fourth (third) quarter, respectively. If the difference is in the fourth quarter, the first 3*k*/4 nucleotides are equal, and we can linearly scan the sorted *k*-mer list, divide it into corresponding buckets of equal 3*k*/4 first nucleotides and run a bit-parallel pairwise Hamming test within each bucket. The overall efficiency of this approach depends on the relation between *n* and $4^{3k/4}$ and not having large buckets. If *k* is sufficiently large, many of the buckets will contain only a few elements, and the process is indeed very fast. It remains to deal with pairs *x*, *y* that differ in one nucleotide in their third quarter. Details are given in Section Pairwise comparisons in small buckets (Quarter).

### Recursive 4-way comparisons (FourWay)

**Fig. 1:**
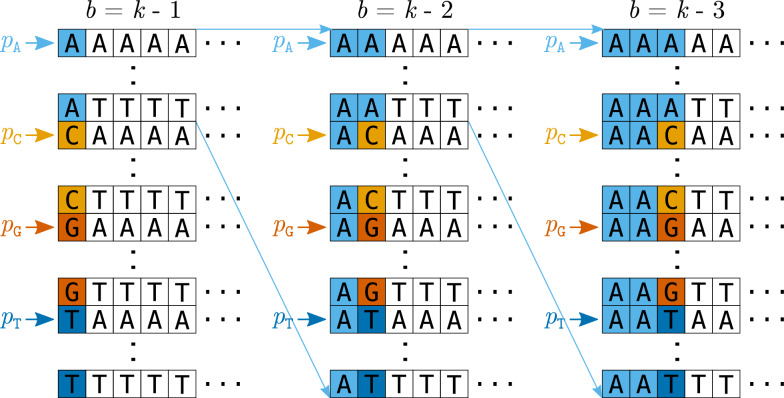
Recursive 4-way comparison at different depths $d=1,2,3$, from left to right. At depth *d*, the first $d-1$ characters of all *k*-mers within the considered interval are equal, and the *d*-th character is compared. Let $b:= k-d$. The (at most) four *b*-mer suffixes of the *k*-mers pointed to by pointers $p_c$, $c\in \Sigma$ are compared, and the minimal *b*-mer(s) are identified among the four elements. The *k*-mers with equal minimal *b*-mers are marked as weak if there are at least two equal minimal *b*-mers. The pointers of minimal *b*-mers are incremented (or inactivated, if their bucket has been completely scanned). After the 4-way comparison of an interval is completed, it is repeated recursively with increased depth $d+1$ on its 4 sub-intervals (only the first such recursive call for the A-subinterval is shown)

**Fig. 2:**
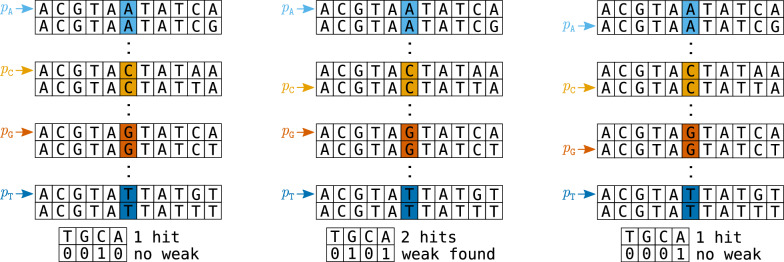
Weak *k*-mer identification by 4-way comparison at depth $d=6$. The first $d-1=5$ characters are identical. Pointers $p_c, c\in \{\texttt {A,C,G,T}\}$, point at *k*-mers whose *d*-th character is *c*. Considering the $b:= (k-d)$-suffixes, we identify the smallest suffix among the pointed-to *k*-mers. Left panel: The smallest suffix is TATAA at $p_\texttt {C}$, as indicated by the bit vector (0010). As a single *b*-suffix is minimal, no weak *k*-mers are identified, and $p_\texttt {C}$ is increased. Middle panel: The smallest *b*-suffix is TATCA at $p_\texttt {A}$ and $p_\texttt {G}$. Therefore, the two *k*-mers pointed to by $p_\texttt {A}$ and $p_\texttt {G}$ are identical except for their *d*-th characters, and we have found a weak pair. Both $p_\texttt {A}$ and $p_\texttt {G}$ are incremented. Right panel: The smallest *b*-suffix is TATCG at $p_\texttt {A}$ only. No weak *k*-mers are identified, and $p_\texttt {A}$ is incremented

### Basic algorithm

The algorithm FourWay works similarly to 4-way merges. The input is a set *K* of canonical *k*-mers, which are “unpacked” into twice as many forward and reverse complement *k*-mers, which are then sorted lexicographically. This yields a sorted *k*-mer array *A* of length *n*.

The algorithm FourWay is recursive. It is called with a depth parameter $d \in \{1,\dots , k-1\}$ and a list of start pointers $q = (q_c)$ with $c\in \Sigma \cup \{\texttt {\$}\}$. Each invocation $\textsc {FourWay}(A, d, q)$ identifies weak *k*-mers in an interval $I = [q_\texttt {A}, q_\texttt {\$}[$ of *A*, where the first $(d-1)$ nucleotides of the contained *k*-mers are equal, and $q_c$ (for $c\in \Sigma$) points to the start of the sub-interval of *I* where the *d*-th nucleotide is *c* (Fig. 1). The sentinel pointer $q_\texttt {\$}$ points beyond the end of the interval *I*. In the initial call, $d=1$ (no common prefix, the first nucleotide is compared), $q_\texttt {A}=0$ points to the first (smallest) element in *A* (with indexing starting at zero) and $q_\texttt {\$}=n$ points past the end of *A*. The initial values of the other pointers $q_c$, $c\in \{\texttt {C,G,T}\}$ are determined by linearly scanning the sorted array once.

First, we create working copies $p=(p_c)$ of $q=(q_c)$ for $c\in \Sigma$. While *q* will stay unchanged, the $p_c$ increase towards larger elements as the algorithm proceeds. Initially, all the $p_c$ pointers are *active*. When $p_c$ moves beyond the end of its sub-interval (i.e., it reaches a $q_{c'}$ for a character $c' > c$, where the sentinel is larger than any character in $\Sigma$), the pointer becomes *inactive*. If only a single pointer or no pointer is active, we are done and proceed to the recursive calls; see below.

In each step, the algorithm examines the *k*-mers at the locations pointed to by the active pointers $p_c$. We call these the *active k-mers*. They jointly have the following properties:

1. Their first $d-1$ characters are equal (true for all *k*-mers in interval *I*),
2. their *d*-th characters are distinct,
3. their suffixes of length $b = k - d$ are arbitrary, but examined in increasing order;
4. they are the smallest *k*-mers in *I* that have not yet been but still may be identified as weak based on a single difference at their *d*-th character.

The comparison is visualized in Fig. 2. We look at the *b*-suffixes of the active *k*-mers and find the smallest one(s). Let $C^*$ be the character set such that exactly the *k*-mers at $p_c$, $c\in C^*$, have the minimal *b*-suffixes among the active *k*-mers. If $|C^*| \ge 2$, we have identified a group of *k*-mers of size $|C^*|$ that differ only at their *d*-th position; hence all of them are marked as weak. (If $|C^*|=1$, nothing happens.) Then, all $p_c$ for $c\in C^*$ are incremented. These steps are repeated until a single (or no) active $p_c$ remains.

After processing interval *I*, if $d < k$, the sub-intervals are processed recursively, so there are $|\Sigma |=4$ recursive calls, each with increased $d \leftarrow d + 1$ (and reduced $b = k - d$). The initial pointers *q* for each subinterval are obtained by a linear scan through the sub-interval. The recursive call is not performed if the length of the subinterval is at most 1, as there is nothing to compare then.

### Implementation on 2*k*-bit encoded integers

The current implementation uses a 2*k*-bit encoded representation of *k*-mers and is restricted to $k\le 31$, leaving at least one bit for marking weak *k*-mers within the 64-bit integer encoding.

In order to compute $C^*$, and to keep track of the smallest *k*-mers, a 4-bit vector *v* is used. Iterating over the active *k*-mers only, if the *b*-suffix is a new minimum (and also initially), a single bit is set in *v*, corresponding to the nucleotide at position *d* (using $\texttt {A}=(0001)_2 = 1$, $\texttt {C}=(0010)_2 = 2$, $\texttt {G}=(0100)_2 = 4$, $\texttt {T}=(1000)_2 = 8$). If another *b*-suffix is equal to the current minimum, the bit corresponding to the character at position *d* is additionally set in *v* (using bitwise or).

We test whether $|C^*| \ge 2$ by checking if the population count of *v* is at least 2, which is done by testing if $v \, \& \, (v-1) \ne 0$.

### Optimizations and parallelization

In principle, the depth *d* is recursively increased up to *k*, so each character in the *k*-mer is checked for being the single different one. However, since the array contains both all forward and all reverse-complement *k*-mers, we identify each pair $\{x,y\}$ with a Hamming distance of 1 twice, both as $\{x, y\}$ and as $\{rc(x), rc(y)\}$. In one case, the difference is in the first half; in the other case, the difference is in the second half of the *k*-mers. Therefore, we could stop the recursion after checking the first half of the *k*-mers, at depth $\lceil k/2 \rceil$.

However, there are advantages in processing only the second half of the *k*-mers instead of the first half, i.e. start at depth $d = \lfloor k/2 \rfloor$ and do recursive steps until the depth *k* is reached. With an increasing depth *d*, the number of elements in an interval decreases until it is reduced to only one or zero element(s). Even before we reach the point at which only one element is left, the book-keeping for the recursion creates more work than the actual comparison of the elements. Therefore, if the interval length drops below a certain value (we evaluate good thresholds in Section ''Interval length thresholds for FourWay+Pairwise''), we switch to the direct bit-parallel pairwise comparison. We call this optimization the FourWay+Pairwise method. Since we process the second half of the *k*-mers, each interval has a fixed constant prefix of length at least $\lfloor k/2 \rfloor$, yielding already relatively short intervals in the first step. We then typically switch to the pairwise comparison after just a few recursions. In practice, this results in a noticeable speedup. To partition the sorted *k*-mer set into blocks with the same length-$\lfloor k/2 \rfloor$ prefix, we do a single linear scan over the whole set of *k*-mers and identify block boundaries where any of the first $\lfloor k/2 \rfloor$ characters change.

For parallelizing the algorithm, we define a prefix length $g \le \lfloor k/2 \rfloor$ to divide the *k*-mers into $4^g$ distinct chunks based on their first *g* characters. By an initial linear scan, we identify the start and end of each chunk and divide them among parallel threads, as each such interval can be processed independently. Inside each chunk, the *k*-mers are processed starting at depth $d = \lfloor k/2 \rfloor$. This allows for almost trivial parallelization among threads. Any number of threads *t* can be combined with any number of chunks $4^g \ge t$. Larger values of *g* lead to smaller chunks and better load balancing over the threads until *g* becomes so large that the scheduling overhead cancels the advantages of the fine-granular jobs. Benchmarks concerning the effect of the number of threads and the choice of *g* can be found in Section Parallelization benchmarks after the overall algorithm benchmarks (Section Benchmarks on the human genome–Benchmarks on artificial data with extremal properties) and benchmarks about the threshold for switching to pairwise comparison (Section 4.3).

### Running time analysis

#### Theorem 1

(Time complexity of FourWay) The running time of basic FourWay is $O(n\,k^2)$, where *n* is the number of *k*-mers in the sorted input array *A*, assuming constant alphabet size. If *k*-mers are integer-encoded, which is possible for $k\in O(\log n)$, the time reduces to *O*(*nk*).

#### Proof

The initial call is FourWay(*A*, *d*, *q*) with the full array of length $|A|=n$, depth $d=1$ and initial start pointers $q=(q_c)_{c\in \Sigma }$. It performs a number of steps that is *O*(*n*) because in each step, at least one of the $|\Sigma |=O(1)$ pointers $p_c$ is incremented. In each step, the smallest of the $|\Sigma |$ length-$b=k-d < k$ suffixes are found, so each step takes *O*(*k*) time, which reduces to *O*(1) if the suffix is represented as a machine word sized integer. Then, we recurse into $|\Sigma |$ subproblems FourWay$(A_c, d+1, q_c)$ for $c\in \Sigma$ of total size $\sum _c\, |A_c| = n$. If *T*(*n*, *k*) denotes the total time, we have

$$\begin{aligned} T(n,k) &= O(nk) + \sum _{c\in \Sigma }\, T(n_c, k-1) \text{ with } \sum_{c\in \Sigma }\, n_c = n\,,\\ T(n,1) &= O(n) \,, \end{aligned}$$

which solves to $T(n,k) = O(nk^2)$. Assuming constant-time comparisons, we obtain

$$\begin{aligned} T(n,k) &= O(n) + \sum _{c\in \Sigma }\, T(n_c, k-1) \text { with } \sum_{c\in\Sigma }\, n_c = n\,, \\ T(n,1) &= O(n) \,, \end{aligned}$$

which solves to $T(n,k) = O(nk)$. $\square$

Overall, the FourWay and FourWay+Pairwise algorithms mainly perform (4-way) linear scans of intervals of the array, and therefore have excellent cache locality. Hardware prefetching takes care of moving required *k*-mers into the CPU caches before they are needed for comparison, so there is very little memory latency and high memory throughput for these algorithms by design.

### Pairwise comparisons in small buckets (Quarter)

**Fig. 3:**
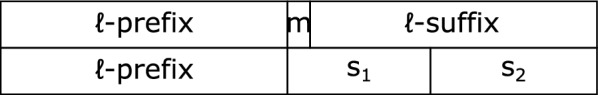
All *k*-mers are split into blocks based on the $\ell := \lfloor k/2 \rfloor$-prefix and further separated into sections based on third quarter $s_1$ and last quarter $s_2$. For odd *k*, the middle position *m* must be included in the third quarter $s_1$. We either have $|s_1|=|s_2|$ or take care that $|s_1|=|s_s|+1$, making the third quarter the longer one if their lengths differ by 1. For example, $25 = 12 + 7 + 6$ with $k=25$, $\ell =12$, $|s_1|=7$ and $|s_2|=6$

**Fig. 4:**
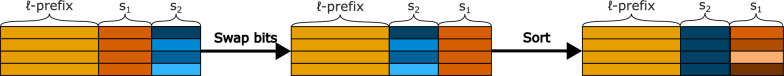
After checking if the *k*-mers differ in a single position in the last quarter $s_2$, existence of a single difference in the third quarter $s_1$ has to be checked. For this, the nucleotides of $s_1$ and $s_2$ are swapped. Then, *k*-mers are locally re-sorted (within buckets of common $\ell = \lfloor k/2 \rfloor$-prefixes), and the algorithm for the last quarter is applied again

### Basic algorithm

With the Quarter algorithm, we reduce the number of pairs for which we calculate the Hamming distance. If two canonical *k*-mers $x\ne y$ have a canonical Hamming distance of $H(x,y)=1$, then at least one of the four pairs from $\{(x, y), (x, rc(y)), (rc(x), y), (rc(x), rc(y))\}$ have their single difference in the third quarter $s_1$ or last quarter $s_2$ of their sequences; in the case of odd *k*, the middle position *m* must be included in the third quarter $s_1$ (see Fig. 3).

If the difference is in the last quarter $s_2$, then pairwise comparisons can be restricted to within buckets that share a common $\ell := \lfloor k/2 \rfloor$-prefix and a common $s_1$ section (for an overall shared 3*k*/4-prefix of the *k*-mer). For example, for $k = 25 = (6+6+7+6)$, we partition the sorted *k*-mer array into intervals that share the same $(6+6+7) = 19$-mers. As already many 19-mers are unique in a mammalian genome, the 25-mer intervals are often small or even contain just a single 25-mer, requiring no further comparisons at all.

If the difference is in the third quarter $s_1$, then pairwise comparisons can be restricted to sets of *k*-mers that share both their $\ell$-prefix and the sequence in the last quarter $s_2$. These sets may be conveniently constructed by local re-sorting within blocks that share a common $\ell$-prefix: Swap the nucleotides belonging to sections $s_1$ with those in $s_2$ and re-sort locally within the $\ell$-block (Fig. 4); then apply the same interval partitioning as above. In the 25-mer example, we would then be using common $(6+6+6) = 18$-mers.

### Implementation and parallelization

To keep track of which *k*-mers are marked as weak, we use one of the 64 bits of the *k*-mers. This limits the implementation to $k \le 31$. For the Quarter algorithm, we use the least significant bit for marking weak *k*-mers. In the sorting step after swapping $s_1$ and $s_2$ it is not necessary to special case the bit. Since it is the least significant bit, the *k*-mers are sorted correctly.

As for FourWay and FourWay+Pairwise, the Quarter algorithm can also be easily parallelized over $4^g$ chunks, where a chunk is defined as the group of *k*-mers that start with a common prefix of length $g \le \lfloor k/2 \rfloor$.

### Pre- and post-processing

#### Optionally chunked input

As presented here, the algorithms start with an already sorted *k*-mer set *K* that contains both forward and reverse-complemented *k*-mers. In practice, one often has an unsorted collection of canonical *k*-mers available, e.g., from a key-value store such as a hash table or file on disk.

However, it may take considerable time and additional memory to convert the available representation into the required input of the algorithms presented here. For example, the *k*-mer counter hackgap can represent the roughly 2.39 billion distinct human canonical 25-mers and counts up to 255 in less than 12 GB of memory, using bit packing and quotienting [12]. However, expanding these into twice as many (4.78 billion) 64-bit integers takes an additional 38 GB of memory (of unpacked data) for identifying the weak 25-mers.

On smaller-memory systems, the input data can be created and processed in smaller chunks (at the expense of speed): Select a small number $s \le \lfloor k/2 \rfloor$ of initial nucleotides; often $s=1$ or $s=2$ is sufficient in practice. Split up the input set *K* into $4^s$ chunks, $K_{j}$, $j=0,\dots ,4^{s}-1$, where chunk $K_j$ is defined as the sorted subset of *K* that has an integer-encoded length-*s* prefix with value *j* (i.e., $enc(x[:s])=j$). Each chunk is generated by linearly scanning over the existing representation and extracting only *k*-mers and reverse complements that start with the correct prefix; this is repeated for each of the $4^s$ chunks. The default case (everything is one big chunk) corresponds to $s=0$. All presented algorithms can be run on each chunk sequentially (and then parallelized based on sub-chunks with more common initial characters).

### Post-processing and result interpretation

The presented algorithms only identify one pair with canonical Hamming distance 1, say $x\ne y$, with their difference in the second half of the sequences, but not the other pair, $rc(x)\ne rc(y)$, with their difference in the first half of the sequences. It is this property that gives us chunks and parallelization essentially for free.

However, this means that post-processing is required. This is typically required anyway, as the weak *k*-mers must be annotated in the original (canonical) representation (say, the hash table). This involves a linear scan of the annotated sorted set *K* and a look-up of the canonical form in the original representation. In the following benchmarks, times for pre- and post-processing are not considered, since they are the same for all approaches.

## Benchmarking results

**Fig. 5:**
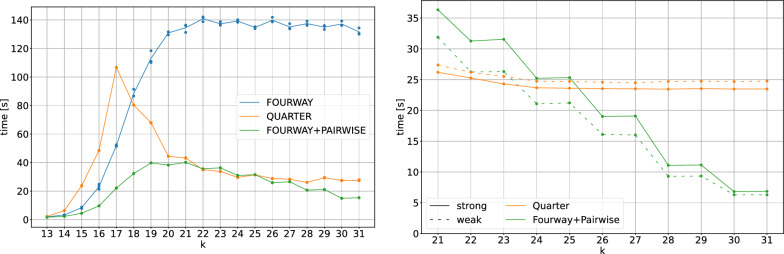
Comparison of the wall times of FourWay, FourWay+Pairwise and Quarter. Left: Wall times for different values of *k* using 8 threads on the t2t human reference genome with varying numbers of *k*-mers depending on *k* (cf. Fig. 8). Shown is the mean (line) over 3 repeated runs (dots). For $k=25$ and 8 threads, the naive neighborhood generation approach needs 3937 s ($\approx 65$ min), while all presented algorithms need less than 140 s, the best ones around 30 s (add 300 s for pre- and post-processing). Right: Wall times for different values of *k* using 8 threads on two artificial datasets with 2 billion *k*-mers each, in which all *k*-mers are either strong (solid lines) or weak (dashed lines)

We evaluate FourWay, FourWay+Pairwise and Quarter on real genomic data and on artificial data with extremal properties. We report wall clock times (“wall times”; real number of seconds passed for weak marking). The benchmarking equipment consists of a workstation with AMD Ryzen 9 5950X 16-core processor with 128 GB of main memory.

Our implementation is written in just-in-time-compiled Python 3.11 using typed numpy arrays [14] and the numba compiler [15], which achieves execution speeds comparable to those of C/C++ implementations. Code is available at https://gitlab.com/rahmannlab/strong-k-mers.

### Benchmarks on the human genome

We compare the running times of the discussed algorithms on the human telomere-to-telomere (t2t) reference genome [16] with roughly 3.1 billion base pairs and 2.5 billion distinct canonical 31-mers, expanded and encoded into approximately 5 billion 64-bit integers, stored in a 40 GB file on disk. Our comparison includes several different values of *k* for the *k*-mer size and different degrees of parallelization with varying numbers of threads.

For benchmarking purposes, we pre-compute the required input array from original genome DNA sequences and read the pre-computed sorted numpy array from disk. This array is pre-computed as follows: We compute the set of all canonical *k*-mers by executing the *k*-mer counter hackgap [12]. This results in an in-memory hash table with all canonical *k*-mers (encoded as 2*k*-bit integers) and their counts. The canonical *k*-mers are expanded to 64-bit integer encodings of the *k*-mers and their reverse complements, and stored in a large uint64 numpy array, which is then sorted and written to disk. The time for this preprocessing step is not included in the measurements reported here.

We compare the running times of our implementations of algorithms FourWay, FourWay+Pairwise and Quarter (Fig. 5 left). As a baseline, the neighborhood generation method (looking up all 3*k* canonical neighbor *k*-mers, or less until the first neighbor is found, for each canonical *k*-mer in the input set; skipping *k*-mers already marked as weak) for $k=25$ using 8 threads needs 3937 s (approximately 65 min), using the multi-way Cuckoo hash table of hackgap [12].

For $k\in \{13,\dots , 31\}$, each algorithm was executed three times; individual wall times (points) and their means (lines) are shown in Fig. 5 (left panel).

The wall time of FourWay correlates with the total number of distinct *k*-mers. For $k\le 22$, the running time increases up to approximately 140 s. The running time for $k\ge 22$ remains nearly constant at 140 s.

The optimization of FourWay+Pairwise, stopping the recursion early if the interval to be examined has at most 24 elements (276 cache-friendly pairwise comparisons), yields a significant reduction of wall time, especially for large *k*. Using 8 threads, the longest wall time is 40 s for $19\le k\le 21$. For longer *k*-mers, the running time decreases down to below 20 s for $k=31$.

The wall time of Quarter increases with the number of distinct *k*-mers for $k\le 17$. Interestingly, the algorithm has its highest running time for $k=17$ with approximately 110 s, even though the number of distinct *k*-mers increases further for larger *k*, while the running time decreases. We here start to see the benefit of longer and more specific 3*k*/4 ($\ge$ 13-mer) prefixes that for increasing *k* yield smaller and smaller buckets for the quadratic-time comparison. If *k* is increased further, this effect is even more noticeable, as the buckets become smaller, while the total number of *k*-mers stays approximately constant. For $k\ge 24$, the wall time is nearly constant at approximately 30 s.

Thus, for $k\ge 24$, Quarter is approximately 4.5 times faster than the purely recursive FourWay approach. However, the optimized FourWay+Pairwise approach, where the recursion stops early and intervals of length at most 24 are examined with the bit-parallel pairwise Hamming distance computation, is even faster than Quarter for $k\ge 28$ and for $k\le 19$ and comparable to Quarter in-between ($20\le k\le 27$).

To compare these times fairly to the baseline neighborhood generation method, one should add 300 s of pre- and post-processing time to the times shown in Fig. 5. Nevertheless, this yields 330 s for Quarter and FourWay+Pairwise against almost 4000 s for neighborhood generation.

### Benchmarks on artificial data with extremal properties

We compare the wall times for Quarter and FourWay+Pairwise on pairs of artificial datasets for different lengths *k*. In one dataset of each pair, all *k*-mers are weak; in the other dataset, all *k*-mers are strong. Both datasets of each pair have a size of 2 billion ($2 \cdot 10^9$) *k*-mers. The datasets are generated in the form of *k*-mers encoded as 64-bit integers, as for the human genome data.

To generate a dataset containing only weak *k*-mers, we first generate a set of random integers between 0 and $4^k$, which make up a quarter of the size of the resulting set. In the next step, we add a Hamming-distance-1 neighbor for each element: We pick one nucleotide and replace it with its complement. After this, we add the reverse complements of all *k*-mers and sort the array. In the last step, we iterate over the sorted array and ensure that all elements are unique. Duplicates are unlikely, given the random generation, and if they happen, we ensure the desired target size even after their removal by initially generating a slightly larger set.

To obtain a dataset that contains only strong *k*-mers, we first generate a slightly larger set than the required size, add the reverse complement of all elements, and remove all duplicates via sorting. In the next step, we run the FourWay+Pairwise algorithm to mark weak *k*-mers and apply post-processing to ensure that the weak *k*-mers are marked as weak in both orientations. We then remove all weak *k*-mers; the remaining array is cut to the desired size and sorted.

The wall times for Quarter and FourWay+Pairwise on both the only weak and the only strong dataset are shown in Fig. 5 (right panel) for different values of *k*, using 8 threads and an interval length threshold of 24 for switching to Pairwise in FourWay+Pairwise.

Perhaps surprisingly, the wall time does not depend strongly on the type of dataset, with only a few seconds difference. We would have expected a longer running time for the weak datasets in comparison to the strong datasets, as marking weak *k*-mers requires writing to memory, whereas this does not happen for the strong datasets. This expected difference can be observed to a small degree for the Quarter algorithm, but for the FourWay+Pairwise algorithm, the relation is reversed, and the weak dataset is processed slightly faster. Overall, the differences are small between the datasets, and may result from different effects, such as the different lengths of the intervals with pairwise comparisons.

The Quarter algorithm’s speed on the artificial data is slightly faster than, but similar to its performance on the human genome data, and essentially constant for all $k\ge 21$. FourWay+Pairwise, however, becomes faster with increasing *k*, and in a more pronounced way than for the human genome data. This can be explained with the structure of the datasets, with the *k*-mers being randomly chosen: For larger *k*, we essentially examine small groups of 2 *k*-mers (weak dataset) or of a single *k*-mer (strong dataset). The number of necessary recursion steps to arrive at such a small cluster drops with *k*. We note that the performance on genome data is more realistic than the one on these artificial datasets.

### Interval length thresholds for FourWay+Pairwise

**Fig. 6:**
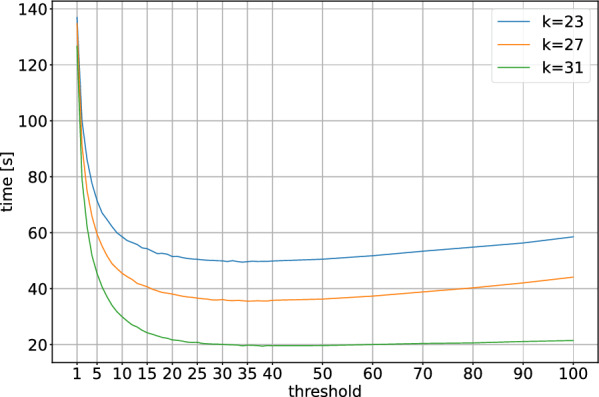
Wall times (using 8 threads) of the FourWay+Pairwise algorithm on the t2t human reference genome with different *k*-mers sizes and thresholds at which we switch to the pairwise comparison

For the FourWay+Pairwise algorithm, an important parameter is the threshold at which we switch from the recursive comparison to the pairwise comparison. In Fig. 6, we evaluate the running time (wall time using 8 threads) of the FourWay+Pairwise algorithm on the t2t-reference for $k\in \{23, 27, 31\}$ using different thresholds. If the threshold is set to 1, we do not use the pairwise comparison at all and continue the recursive approach until we obtain intervals with a single element or empty intervals. In this case, we need between 120 and 140 s, depending on *k*. Even a small increase of the threshold above 1 reduces the running time drastically: A threshold of 10 more than halves the running time. The optimal threshold appears to be between 20 and 40 for $k=23$, 30 and 50 for $k=27$, and 30 and 70 for $k=31$. Overall, a threshold of roughly 30 seems to be best for a wide range of *k*, and the exact choice is less important as long as the threshold is not chosen too small.

### Parallelization benchmarks

**Fig. 7:**
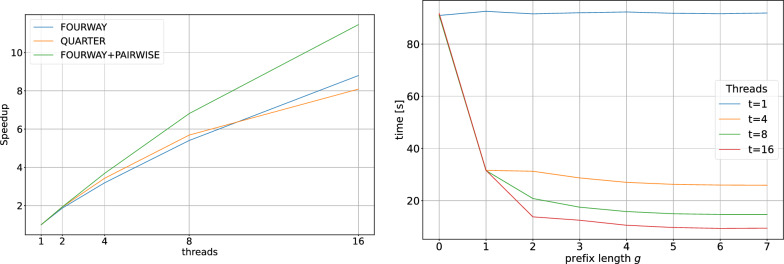
Comparison of the wall time of FourWay, FourWay+Pairwise and Quarter with different parallelization strategies. Left: Speedup as a function of the number of threads used for parallelization, for fixed $k=25$. Right: Wall times for different prefix lengths *g* (x-axis) and number of threads (color). Each choice of $g\le \lfloor k/2\rfloor$ divides the *k*-mers into $4^g$ chunks that can be processed independently in parallel. With $g=0$, the whole array is processed as one chunk and only one thread is used. If $4^g$ is less than the number of threads, only $4^g$ threads can be used

We examine the effect of parallelization for an increasing number of threads (Fig. 7 left), using the t2t reference genome with $k=25$ and 1, 2, 4, 8 and 16 threads. We compute the speedup for *T* threads as usual, dividing the time used by a single thread by the time used by *T* threads, which ideally would give a ratio of *T*. We see that for two threads, the speedup is nearly the desired factor of 2 for all algorithms. For $T=4$, the speedup for Fourway and Quarter is closer to 3 than to 4, but for FourWay+Pairwise, it reaches almost 4. For 8 threads, the result is similar: a speedup of roughly 6 emerges for Fourway and Quarter, but almost 7 for FourWay+Pairwise. The effect is even more pronounced for 16 threads, where Quarter achieves a speedup of 8, FourWay a speedup of nearly 9, but FourWay+Pairwise yields a speedup of almost 12. Overall, the parallelization scales quite well for all algorithms, with a distinct advantage for FourWay+Pairwise.

We also evaluated the effect of different prefix lengths *g* to define chunks that are submitted as independent jobs. For a given value of $g\ge 0$, there are $4^g$ chunks; each chunk contains *k*-mers that start with the same length-*g* prefix (see algorithm descriptions). With $g=0$, there is no parallelization. For this benchmark, we used the human t2t reference genome with $k=25$ and 1, 4, 8 and 16 threads. Figure 7 (right) shows the resulting wall times. Once we can use all 16 threads ($g\ge 2$), a further increase of *g* has a small but beneficial effect on the running time because of the more fine-granular load balancing of more chunks (4096 for $g=6$ or $16\,384$ for $g=7$) between the threads. For further increasing values of *g* (even from $g=6$ to $g=7$), no further benefit is obtained, and the administrative costs of managing the jobs grow exponentially. It seems advisable to use $g=5$, or $g=6$ on large datasets with 16 threads.

## Uniqueness within the human genome

We apply our algorithms to the human telomere-to-telomere reference genome [16] and compute the fraction of weakly unique, strongly unique and non-unique *k*-mers in the whole genome for different values of *k*. We then check whether the distribution of the different 25-mer types is different in regions with genes and in exons. We finally examine the local distribution of the three uniqueness categories across all chromosomes for 25-mers.

### Whole genome statistics

**Fig. 8:**
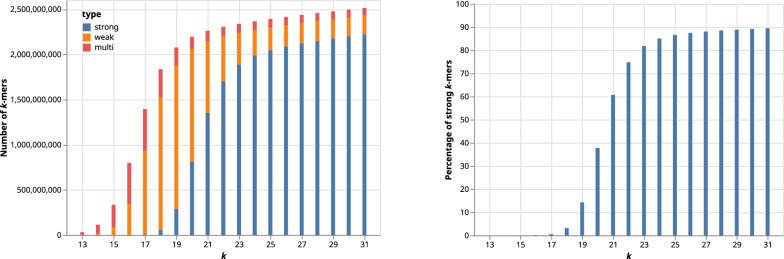
Left: Absolute number of strongly unique, weakly unique and non-unique (multi) *k*-mers in the t2t reference genome for different values of *k* (stacked bar chart). The total number of *k*-mers rapidly increases for *k* between 13 and 19. For $k\ge 21$, there are only a few more new *k*-mers with increasing *k*. Right: Percentage of strongly unique *k*-mers among the distinct *k*-mers. For $k > 25$, almost 90% of the distinct *k*-mers are strong

As shown in Fig. 8, the total number of distinct *k*-mers in the t2t reference genome increases with increasing *k*. Since the t2t reference genome contains $\approx 3.1$ billion nucleotides, for $k\le 15$, most of the approximately $4^k / 2$ canonical *k*-mers are present in the DNA sequence. Consequently, almost all of these *k*-mers are weakly unique or occur multiple times. For $k\ge 18$, the *k*-mers get more unique and specific, and the increase of distinct *k*-mers in the sequence flattens out for $k\ge 21$. At the same time, the number of strong *k*-mers increases. For $k=18$, only approximately $4\%$ of the *k*-mers are strong. For $k=23$, already more than $80\%$ of the *k*-mers are strong. For $k\ge 24$, the number of strong *k*-mers gradually approaches 90% of the distinct *k*-mers.

### Uniqueness in genes and exons

**Fig. 9:**
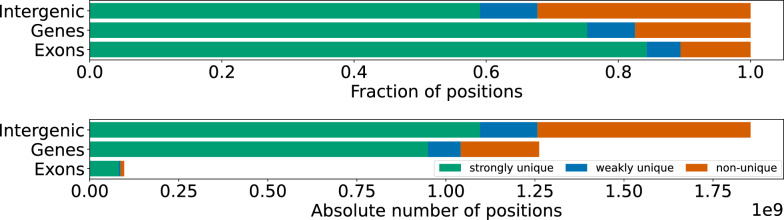
Comparison of 25-mers in intergenic regions, coding genes and exons. A 25-mer is part of a gene/exon if it is completely contained in the corresponding annotated interval. Top plot: relative fractions. Bottom plot: absolute number of positions

In Fig. 9, we examine how 25-mers are classified in intergenic regions, in gene regions, and specifically in exons. A *k*-mer is considered to be part of a gene or an exon, if it is completely contained in the corresponding interval (starts and ends in the gene or exon, respectively). The intervals are extracted from the annotation file of the t2t reference genome [16]. For the genes, we have limited the selection to protein coding genes by extracting only genes with the attribute protein_coding. The genes are the complete intervals annotated as genes, including the coding exons, but also the untranslated 5’ and 3’ regions and (sometimes very long) introns. The intergenic region is the remaining part of the genome after excluding the genes.

Figure 9 shows that most of the t2t-reference-genome consists of intergenic regions. In these regions, we have the highest proportion of non-unique *k*-mers. Within the genes, the fraction of strongly unique *k*-mers is at about 75%. When focusing on protein coding exons, the fraction of strongly unique *k*-mers is $>80\%$. This enables applications with a focus on exons to be based on strongly unique *k*-mers. For example, the typical (protein-coding) gene should be uniquely identifiable by a large number of strongly unique 25-mers.

### Fine-grained uniqueness distribution

**Fig. 10:**
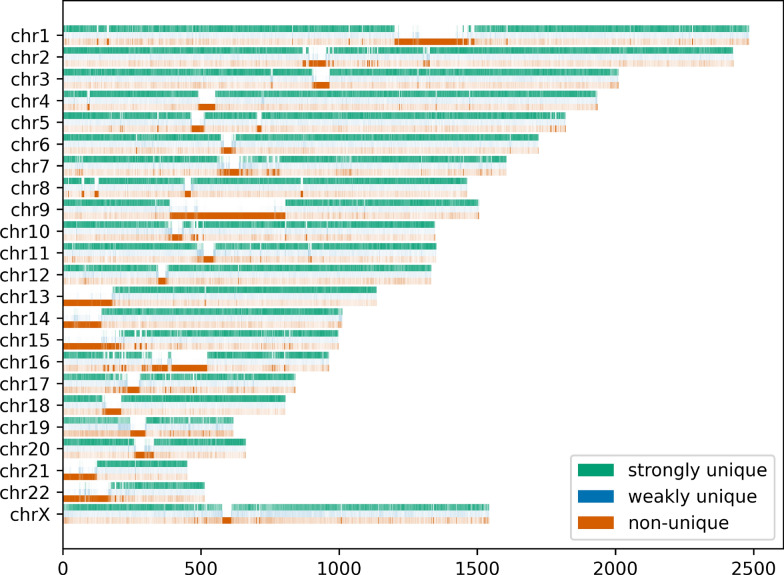
Distribution of strongly unique, weakly unique and non-unique *k*-mers in the t2t reference genome. We split the genome into blocks of 100 000 base pairs. For each block, we computed the relative fraction of strongly unique (green), weakly unique (blue) and non-unique (orange) *k*-mers that start in it. Each block is represented by a small vertical stroke; the relative fractions are encoded by the transparency from 0.0 (invisible) to 1.0 (solid color). Regions with repetitive DNA become visible, in particular the centromeres, but also other repeats

We have taken a closer look at which positions strongly unique 25-mers are present in the human genome. In Fig. 10, we visualize the distribution of the different types of 25-mers in the reference genome, excluding the Y chromosome and the mitochondrial DNA, at a resolution of 100 000 basepair blocks.

As expected, the centromeres of all chromosomes mainly consist of non-unique *k*-mers. The strongly unique *k*-mers (green) are spread over all chromosomes. Weakly unique *k*-mers are rare in general but can also be found in most parts of the chromosomes, but are concentrated around the centromeres and in the X chromosome.

## Application perspectives

Among the many possibilities, we discuss two possible applications of strongly unique *k*-mers that are enabled by the new efficient algorithms to identify weak *k*-mers presented in Section Methods: sequencing error correction and xenograft sorting.

### Sequencing error correction

**Fig. 11:**
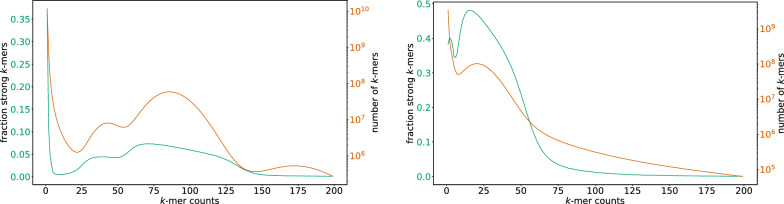
25-mer frequency histogram (orange right y-axis; log-scale) and fraction of strong 25-mers (green left y-axis; linear scale) for two datasets. Left panel: An Ossabaw pig with a high average coverage of $80\times$. A high number of the distinct 25-mers in this sample is unique. Of these, 37% are strongly unique and 63% are weakly unique. Right panel: The son of the GIAB Ashkenazi Trio (HG002). The average coverage is $25\times$. Again, a high number of the distinct *k*-mers in this sample is unique. Of these, 39% are strongly unique and 61% are weakly unique

Different types of errors can occur during DNA sequencing. While insertions and deletions are rather rare, substitutions are the most common, at least for typical high-throughput sequencing technologies [17, 18]. Several approaches to correct reads that may contain sequencing errors are based on *k*-mer frequencies in the sequenced dataset. If a read includes a sequencing error, all of the *k*-mers that overlap the erroneous position are changed. As these errors are rare, the same error typically does not happen twice or at least not very often; this is a common assumption. Therefore, sequencing errors are expected to generate a number of unique or rare *k*-mers in the sequenced dataset that have a Hamming distance of 1 to a much more frequent (actually present) *k*-mer in the dataset.

Error correction methods may exploit this as follows: For each unique or rare *k*-mer *x*, one may ask whether there is a single frequent Hamming-distance-1 neighbor *y* in the dataset. If several such rare *k*-mers overlap the same basepair in a read and agree on the same basepair in the respective frequent neighboring *k*-mers, the read can be reliably corrected at this position. Our algorithms would need to be modified to report the pairs (*x*, *y*) if one wanted to use them for error correction, but they are good candidates to speed up existing error correction tools [19, 20

However, we were wondering if the usual assumption that most rare *k*-mers are Hamming neighbors of frequent *k*-mers is actually true, as this is an assumption of many error correction methods, and has, to our knowledge, never been systematically examined. One would expect that almost all of the rare *k*-mers (especially unique *k*-mers) in a whole genome sequencing (WGS) dataset are weak, and that there are few strongly unique *k*-mers.

We therefore applied the FourWay+Pairwise method to the 25-mers of two WGS datasets. The first one is from an Ossabaw pig (SRA accession SRR19175873) with a high mean coverage of $80\times$. The second one are the NIST_Stanford_Illumina_6kb_matepair reads of the son of the GIAB Ashkenazi Trio (HG002). For each dataset, the 25-mer frequency histogram (orange y-axis; log scale) and the fraction of strong 25-mers (green y-axis; linear scale) are overlaid in Fig. 11 for every k-mer occurrence count up to 200.

In the high-coverage dataset (left, $80\times$ coverage), the fraction of strong 25-mers is always below 10%, except for rarely occurring 25-mers, and especially unique 25-mers, where is rises up to 37%. This is an unexpectedly high fraction if we assume that the unique and rare 25-mers are erroneous versions of the frequent ones. Nonetheless, 63% of the unique 25-mers are weakly unique and can potentially be used for error correction. It is unclear where the strong rare 25-mers originate from: Are they modified versions of more frequent 25-mers derived by an insertion or deletion, which we do not detect, or are they modified by more than a single substitution? Do they originate from non-human DNA (contamination), or from a different unknown source?

For the standard-coverage dataset (Fig. 11 right, $25\times$), the picture is different, and a substantial fraction (between 40% to 50%) of frequent *k*-mers with their occurrence frequency around the main peak of 25 is strong, i.e., without a Hamming-distance-1 neighbor in the dataset. Interestingly, of the very rare and unique *k*-mers, also a large fraction of close to 40% is strong, which is a similar fraction as in the high-coverage dataset.

These results suggest that a close investigation of the *k*-mer dynamics in WGS datasets may be warranted, especially concerning commonly made assumptions for error correction.

### Xenograft sorting

The concept of weakly unique and strongly unique *k*-mers can be adjusted to different applications by slightly modifying the definition of *unique*. When indexing a reference genome, as exemplified in our results, a *k*-mer is naturally unique if it occurs at a single position on a single chromosome, considering both strands of all chromosomes.

However, in a transcript quantification task, it should be considered as unique if it occurs in a single transcript, but it may occur several times in that transcript. In a metagenomics species identification or quantification task, it should be considered as unique if it occurs in a single species (possibly several times).

As a concrete example, we now have a closer look at the xenograft sorting problem briefly mentioned in the Introduction. The aim is to classify a set of reads according to their species of origin (e.g., human tumor or mouse tissue). The alignment-free xenograft sorting tool xengsort [3] initially builds an index of all canonical 25-mers in the human and mouse reference genome and associates species information (only human, only mouse, both) with each canonical 25-mer. In this application, a *k*-mer is unique if it is only part of one species (human or mouse, but possibly occurs several times in the same species). A *k*-mer is marked as weakly unique if it is unique for human [mouse] but has a Hamming-distance-1 neighbor that occurs in mouse [human], or in both species.

For marking weak *k*-mers in this application, our algorithms have to be modified to take the associated species information (“values”) into account: Even if a neighboring *k*-mer $x'$ exists for some *k*-mer *x* in the *k*-mer set of human and mouse, the pair $x, x'$ would only be considered as weak if they were part of different species (or one of them part of both species).

The published xengsort application contains such a method, which is run on the union of the genomes, containing $4\,496\,607\,845 \approx 4.5$ billion 25-mers. As reported in the original publication [3], this takes 158 wall-time *minutes* overall, even though it is at least partially parallelized with 8 threads. This reported time on a 64 GB machine does include severaled chunk passes through the array and pre- and post-processing.

We have replaced this method in xengsort by $\textsc {Quarter}$ and run it under the same circumstances on the same machine, with the same number of passes, the same number of threads, and with the same pre- and post-processing. The total time using the Quarter algorithm is 6.1 min instead of the original 158 min, a speedup factor of 25.9. This illustrates the potential of our new algorithms for real-world applications in DNA sequence analysis.

## Discussion and conclusion

We have introduced the weak (canonical) *k*-mer identification problem, which does not seem to have been considered at a large scale elsewhere, even though the identification of strongly unique *k*-mers is useful in many different contexts in alignment-free sequence analysis. We have developed and evaluated engineered algorithms to identify weak *k*-mers that require a sorted array of *k*-mers and their reverse complements as input. The best method FourWay+Pairwise needs at most 40 s to identify weak *k*-mers for any $k=13,\dots ,31$ on the human t2t reference genome, using 8 threads. All methods need less than 2.5 min for each of these tasks. These times are much faster than for querying all 3*k* canonical neighbors for each canonical *k*-mer in a fast hash table (65 min for 25-mers on the same dataset), even when adding the initial sorting time for the *k*-mer array (below 300 s).

The FourWay recursive comparison based algorithm benefits from its cache-friendly design and from hardware prefetching, but suffers from book-keeping overhead once the examined intervals become small. The improved FourWay+Pairwise method that switches to pairwise Hamming distance tests on small intervals is always faster, for any reasonable interval length threshold to switch to pairwise comparisons. The Quarter grouping algorithm shows comparably good performance to FourWay+Pairwise for $k\ge 21$, but suffers from large buckets for smaller *k*. Overall, FourWay+Pairwise is the method of choice with overall best performance and excellent parallelization performance.

The new algorithms have practical relevance in applications, as shown by the the xenograft sorting example in Section Xenograft sorting. Moreover, our results on the classification of rare and frequent *k*-mers in WGS datasets suggest further research to better understand potential improvements for error correction methods on raw DNA sequence data.

From a practical point of view, it is of high interest to develop methods that do not require a sorted expanded array of *k*-mers as input but that instead work directly on a compact hash table representation of the input set, similar to neighborhood generation, but with performance comparable to the methods presented here.

The algorithms in this work have been engineered for a Hamming distance of 1. It would be interesting to extend the problem for Hamming distances $\ge 2$, as this would increase the tolerance against a combination of sequencing errors and SNPs in a small interval. However, one would most likely need a different approach, perhaps a transformation to eulertigs [21] and branching on their FM index, or an approach based on search schemes [22].

## Data Availability

No datasets were generated or analysed during the current study.

## Code Availability

See https://gitlab.com/rahmannlab/strong-k-mers.
